# Effect of advanced nursing care on psychological condition in patients with chronic heart failure: A protocol of systematic review and meta-analysis

**DOI:** 10.1097/MD.0000000000020355

**Published:** 2020-06-05

**Authors:** Yang Lin, Zhi-qiang Su, Shan-shan Yu

**Affiliations:** a Department of Respiratory and Critical Medicine; b Department of Hyperbaric Medicine; c Department of Cardiothoracic Surgery, First Affiliated Hospital of Jiamusi University, Jiamusi, China.

**Keywords:** advanced nursing care, chronic heart failure, effect, psychological condition

## Abstract

**Background:**

This study will appraise the effect and safety of advanced nursing care (ANC) on psychological condition (PC) in patients with chronic heart failure (CHF).

**Methods:**

The following databases will be sought from the beginning up to the February 29, 2020: MEDLINE, EMBASE, Cochrane Library, Web of Science, Scopus, the Cumulative Index to Nursing and Allied Health Literature, the Allied and Complementary Medicine Database, the Chinese Scientific Journal Database, and China National Knowledge Infrastructure. There are not language and publication status limitations related to any electronic databases. In addition, we will also identify conference proceedings, reference lists of included studies, and websites of clinical trials registry. Two reviewers will separately carry out study selection, data extraction, and study quality evaluation. Any inconsistencies will be solved by a third reviewer through discussion. RevMan 5.3 software will be utilized to carry out statistical analysis.

**Results:**

This study will comprehensively summarize all potential evidence to systematically address the effects and safety of ANC on PC in patients with CHF.

**Conclusion:**

The findings of the present study will help to determine whether ANC is effective or not on PC in patients with CHF.

**Study registration number:**

INPLASY202040077.

## 1 Introduction

Chronic heart failure (CHF) is a very common cardiovascular disease, caused by structural or functional cardiac abnormalities.^[1–4]^ It is estimated that the incidence is more than 4% and 1-year mortality is more than 20% in the elder patients with CHF; and its prevalence is more than 20% in patients older than 75 years old.^[5–7]^ Many patients with CHF experience psychological condition (PC) (including depression and anxiety) with at least prevalence of 20%.^[8–16]^ Despite a variety of studies reported that advanced nursing care (ANC) can benefit patients with PC,^[17–30]^ no systematic review has been published to address the effects and safety of ANC on PC in patients with CHF. Thus, this systematic review will appraise the effects and safety of ANC on PC in patients with CHF.

## 2 Methods

### 2.1 Objectives

This study will comprehensively address the effect and safety of ANC on PC in patients with CHF.

### 2.2 Study registration

This study has been registered on INPLASY202040077, and it has been conducted according to the guidelines of Preferred Reporting Items for Systematic Reviews and Meta-Analysis (PRISMA) Protocol statement.^[31]^

### 2.3 Eligibility criteria for study selection

#### 2.3.1 Types of studies

This study will include all potential randomized controlled trials (RCTs) that investigated the effect and safety of ANC on PC in patients with CHF. We will exclude any other types of studies, such as animal studies, reviews, non-clinical trials, and uncontrolled trials.

#### 2.3.2 Types of patients

All patients who were diagnosed as CHF with PC will be included in this study, in spite of their country, sex, age, and duration or severity of CHF and PC.

#### 2.3.3 Types of interventions

In the experimental group, all patients utilized ANC to manage PC.

In the control group, no limitations were applied to the control treatments. However, we will not consider study involved control treatment with any forms of ANC.

#### 2.3.4 Types of outcomes

The primary outcomes are depression (as assessed by any associated scales, such as Hamilton Depression Rating Scale), and anxiety (as evaluated by any relevant tools, such as Hamilton Anxiety Rating Scale).

The secondary outcomes are all-cause mortality, urine output, change in serum sodium, health related quality of life (as measured by any related scores, such as 36-Item Short Form Health Survey), and adverse events.

### 2.4 Search strategy for identification of studies

We will search the electronic databases from the beginning up to the February 29, 2020: MEDLINE, EMBASE, Cochrane Library, Web of Science, Scopus, the Cumulative Index to Nursing and Allied Health Literature, the Allied and Complementary Medicine Database, the Chinese Scientific Journal Database, and China National Knowledge Infrastructure. No language and publication status limitations will be applied to all electronic databases. The details of search strategy for Cochrane Library will be created (Table 1). We will adapt similar strategies to other electronic databases.

**Table 1:**
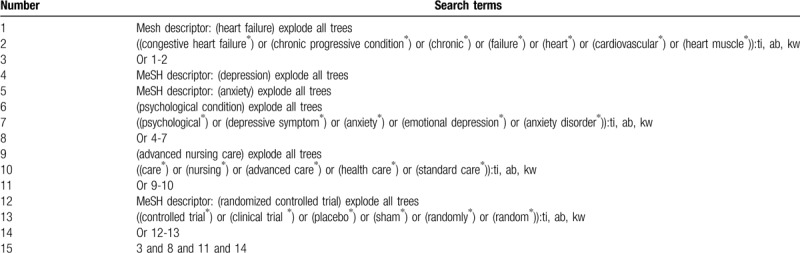
Search strategy of Cochrane Library.

In addition, we will examine conference proceedings, reference lists of included studies, and websites of clinical trials registry.

### 2.5 Study selection

We will utilize EndNote 7.0 software to manage all retrieved citations. Two independent reviewers will identify titles/abstracts of all citations, and duplicates and irrelevant records will be excluded. Then, full-text of potential articles will be obtained and read against all eligibility criteria. Any confusion over inclusion criteria will be determined by a third review through discussion. Details of study selection will be summarized in a PRISMA flow diagram.

### 2.6 Data extraction

Two reviewers will extract data from each eligible trial separately using previous defined data extraction sheet. Any different opinions will be solved by discussion with a third reviewer. The extracted data includes general information (e.g., first author, year of publication), trial methods (e.g., trial design, trial duration, trial setting), participants (e.g., race, gender, age, severity of PC, and CHF), and intervention and controls (e.g., modality types, duration, frequency), outcomes, safety, funding sources, and conflict of interest.

### 2.7 Missing data dealing with

We will contact original authors to request any missing or unclear data when we identified it. If we can not obtain such data, we will just analyze available instead.

### 2.8 Risk of bias assessment for eligible trials

Two reviewers will appraise risk of bias for each included article using Cochrane Risk of Bias Tool through 7 domains. Each 1 is graded as low, unclear, or high risk of bias. All different views will be resolved by a third reviewer through discussion.

### 2.9 Data synthesis

We will employ RevMan 5.3 for statistical analysis. All continuous values will be estimated by mean difference or standardized mean difference and 95% confidence intervals (CIs), and all dichotomous values will be calculated by risk ratio and 95% CIs. *I*^*2*^ statistic will be utilized to test heterogeneity among included trials. *I*^*2*^ ≤ 50% means homogeneity, and a fixed-effect model will be applied. *I*^*2*^ > 50% implies obvious heterogeneity, and a random-effect model will be placed. If possible, we will perform a meta-analysis. Otherwise, we will carry out a subgroup analysis to investigate sources of considerable heterogeneity. If we can still identify significant heterogeneity after subgroup analysis, a meta-analysis will not be conducted. Instead, a systematic narrative synthesis for study findings will be carried out.

### 2.10 Subgroup analysis

We will employ a subgroup analysis according to the differences in study characteristics, study quality, and different types of interventions and outcomes.

### 2.11 Sensitivity analysis

We will undertake a sensitivity analysis to examine the robustness and stability of study findings by eliminating low quality trials.

### 2.12 Reporting bias

We will conduct a funnel plot^[32]^ and an Egger regression test^[33]^ to examine reporting bias when at least 10 trials are included.

### 2.13 Ethics and dissemination

This study will not obtain individual data, thus, no research ethics approval is required. The findings of this study will be published in a peer-reviewed journal or conference presentations.

## 3 Discussion

This study aims to analyze the effect and safety of ANC on PC in patients with CHF. We will apply rigorous methodology to examine studies reporting the PC outcomes of ANC for patients with CHF. Although most studies have reported the effect and safety of ANC on PC in patients with CHF, no systematic review regarding this issue has been published. Therefore, exploring a systematic review of this topic is very necessary and important. This study will provide current evidence on the effect and safety of ANC on PC, which may benefit practitioners, patients, and policymakers.

## Author contributions

**Conceptualization:** Yang Lin, Zhi-qiang Su, Shan-shan Yu.

**Data curation:** Yang Lin, Shan-shan Yu.

**Formal analysis:** Yang Lin, Zhi-qiang Su, Shan-shan Yu.

**Funding acquisition:** Yang Lin.

**Investigation:** Yang Lin.

**Methodology:** Zhi-qiang Su.

**Project administration:** Yang Lin.

**Resources:** Zhi-qiang Su, Shan-shan Yu.

**Software:** Zhi-qiang Su, Shan-shan Yu.

**Supervision:** Yang Lin.

**Validation:** Yang Lin, Zhi-qiang Su, Shan-shan Yu.

**Visualization:** Yang Lin, Shan-shan Yu.

**Writing – original draft:** Yang Lin, Zhi-qiang Su, Shan-shan Yu.

**Writing – review & editing:** Yang Lin, Zhi-qiang Su, Shan-shan Yu.
